# Children’s Neural Sensitivity to Prosodic Features of Natural Speech and Its Significance to Speech Development in Cochlear Implanted Children

**DOI:** 10.3389/fnins.2022.892894

**Published:** 2022-07-12

**Authors:** Yuebo Chen, Qinqin Luo, Maojin Liang, Leyan Gao, Jingwen Yang, Ruiyan Feng, Jiahao Liu, Guoxin Qiu, Yi Li, Yiqing Zheng, Shuo Lu

**Affiliations:** ^1^Department of Otolaryngology, Sun Yat-sen Memorial Hospital, Sun Yat-sen University, Guangzhou, China; ^2^Department of Chinese Language and Literature, The Chinese University of Hong Kong, Hong Kong, Hong Kong SAR, China; ^3^School of Foreign Languages, Shenzhen University, Shenzhen, China; ^4^Neurolinguistics Teaching Laboratory, Department of Chinese Language and Literature, Sun Yat-sen University, Guangzhou, China; ^5^Department of Neurology, The Third Affiliated Hospital of Sun Yat-sen University, Guangzhou, China; ^6^Department of Clinical Neurolinguistics Research, Mental and Neurological Diseases Research Center, The Third Affiliated Hospital of Sun Yat-sen University, Guangzhou, China; ^7^Hearing and Speech Science Department, Guangzhou Xinhua University, Guangzhou, China

**Keywords:** natural speech perception, prosodic feature, neural response, cochlear implantation, speech communication ability, temporal cortex

## Abstract

Catchy utterances, such as proverbs, verses, and nursery rhymes (i.e., “No pain, no gain” in English), contain strong-prosodic (SP) features and are child-friendly in repeating and memorizing; yet the way those prosodic features encoded by neural activity and their influence on speech development in children are still largely unknown. Using functional near-infrared spectroscopy (fNIRS), this study investigated the cortical responses to the perception of natural speech sentences with strong/weak-prosodic (SP/WP) features and evaluated the speech communication ability in 21 pre-lingually deaf children with cochlear implantation (CI) and 25 normal hearing (NH) children. A comprehensive evaluation of speech communication ability was conducted on all the participants to explore the potential correlations between neural activities and children’s speech development. The SP information evoked right-lateralized cortical responses across a broad brain network in NH children and facilitated the early integration of linguistic information, highlighting children’s neural sensitivity to natural SP sentences. In contrast, children with CI showed significantly weaker cortical activation and characteristic deficits in speech perception with SP features, suggesting hearing loss at the early age of life, causing significantly impaired sensitivity to prosodic features of sentences. Importantly, the level of neural sensitivity to SP sentences was significantly related to the speech behaviors of all children participants. These findings demonstrate the significance of speech prosodic features in children’s speech development.

## Introduction

Catchy utterances, such as proverbs (i.e., “No pain, no gain” in English), verses, and nursery rhymes, contain strong-prosodic (SP) features and are child-friendly in speech repeating and memorizing (Yuzawa and Saito, 2006). Prosodic features can be recognized by the variation in pitch, loudness, and duration (Everhardt et al., 2022) and play an important role in children’s speech development. Behavioral studies found successful prosody perception facilitated children’s speech acquisition in that they used speech prosodic information to segment words (Jusczyk et al., 1999; Johnson and Jusczyk, 2001), discriminate emotion (Scheiner et al., 2006; Flom and Bahrick, 2007), and eliminate syntactic ambiguity (Snedeker and Yuan, 2008). Considering that childhood is the critical period of neural plasticity and that neural function development for speech prosody perception can be highly related to both biological growth and the environment (Werker and Hensch, 2015), the correlation between speech development and neural functional development for speech prosody perception in children is worth investigating.

However, neither the underlying neural mechanism of children’s prosody perception nor its specific relationship with children’s speech development has been studied extensively. One possible reason is that the widely used neural functional imaging technique, such as functional magnetic resonance imaging (fMRI) is noisy and highly sensitive to motion artifacts, hence is particularly not applicable for young children (Soltanlou et al., 2018). Functional near-infrared spectroscopy (fNIRS), however, is well accepted as a child-friendly optical neuroimaging technique (Quaresima et al., 2012; Saliba et al., 2016). A few fNIRS studies have explored single acoustic aspects of speech prosody for children, such as rhythm perception (Kovelman et al., 2012), intonation perception (Arimitsu et al., 2011), and prosodic emotion perception (Grossmann et al., 2010), which identified the active role of regions in the right hemisphere. Besides the limited number of studies, another limitation in previous studies is that most studies used single words or artificial utterances to test the neural processing of prosodic features. Little is done targeting directly the actual catchy utterances being used in daily life. In this study, we postulated that there is a specific neural sensitivity in normally developing children for their perception of catchy speeches, and furthermore, such sensitivity facilitates speech development as children’s speech-related neural network development benefits from it. Thus, we expected that the neural responses in perceiving SP sentences would somehow associate with children’s speech communication abilities.

Studies of sensory loss can provide a model for understanding the mechanism of neural function development. With respect to pre-lingually deaf children, the maturation of the auditory cortex (Knudsen, 2004; Ni et al., 2021) and their speech development (Venail et al., 2010) have been influenced due to hearing deprivation. Although cochlear implantation (CI) has become widely used to restore severe-to-profound deafness for pre-lingually deaf children (Sharma et al., 2015), the cochlear device cannot accurately deliver all kinds of sound information, especially prosodic features, possibly because of its limited number of electrodes (Svirsky, 2017). Behavior studies found CI users had difficulty in perceiving pitch changes (Gfeller et al., 2007; Jung et al., 2010), especially in the higher frequency range and discriminating intonation and tones (Peng et al., 2012), which are all related to the speech prosody. Their impaired ability to perceive low frequency pitch changes was also identified and found to be correlated with the overall speech rehabilitation outcomes. Adult CI users were also found to have lower accuracy in discriminating word stress, vowel length, compound words, or phrases (Morris et al., 2013). Nevertheless, a study found that sentences with stronger prosodic features were easier for adult CI users to understand and repeat, which implies the importance of natural catchy sentences for speech communication abilities (Aarabi et al., 2017).

By so far, little is known about the neural processing characteristics of natural catchy sentences with SP features, especially in pre-lingually deaf children after CI. Given the idea that cortical development plays an important role in speech development in pre-lingually deaf children after CI (Liang et al., 2014; Sharma et al., 2015), detecting the neural dysfunction corresponding to SP speech perception in children with CI can offer a window to identify the developmental characteristics of the neural sensitivity to speech prosody and the possible relevance to speech acquisition and shed light on speech development. We then raised the second hypothesis of this study that CI children had characteristic impairments in perceiving the strong prosodic features in catchy speeches, which probably correlates with their speech development.

This study made use of both the child-friendly and CI-safe neuroimaging technique of fNIRS to explore the neural functional characteristics in perceiving SP sentences, and the relation between neural responses and children’s speech communication ability. We expected that SP sentences would induce significantly stronger activation in the right temporal area compared to a weak-prosodic (WP) sentence in normal hearing (NH) children. We also identified CI children’s idiosyncratic deficits in the neural sensitivity to SP features by comparing them with the NH group. Last but not the least, we anticipated correlations between neural responses to SP features and speech communication ability for the participants, which might motivate future studies on the significance of catchy sentences in children’s speech functional neural development.

## Materials and Methods

### Participants

Twenty-five Chinese NH children belonging to the NH group and twenty-two Chinese children with unilateral CI in the right ear were enrolled in this study, whose native language is Mandarin Chinese. In this study, the age of the NH group ranged from 5 years and 1 month (indicated henceforth as 5; 1) to 7; 8, with an average age of 6 years, while the age of the CI group ranged from 5; 0 to 10; 9, with an average age of 6; 8 years old. We intended to explore the possible link between speech prosody perception and speech communication development. It is reported that by 5 years old, children obtain basic oral speech communication ability and begin to develop social communication skills (Eisenberg et al., 1993; Kostelnik et al., 2014). Therefore, we included children older than 5 years in this study. As for children with CI, it was reported that speech improves rapidly through the first 12–18 months after CI (Martines et al., 2013). As a result, we chose children with CI who were older than 5 years and had CI experience of more than 12 months. As children with CI might have delayed speech communication development compared to their peers, we extended the upper limit but kept the average age roughly the same.

All children with CI are pre-lingually deaf. One child with CI was excluded due to an insufficient number of completed trials. The remaining twenty-one CI subjects and all NH participants were right-handed as confirmed by the Edinburgh Handedness test (Oldfield, 1971) and had no history of neurological illness. All participants were native Chinese speakers with no neurocognitive or motor impairments and had a normal or corrected-to-normal vision. All NH participants had no known hearing problems and passed a pure tone audiometry air-conduction hearing screen performed at 0.5, 1, 2, and 4 kHz at 20 dB HL in both ears [test adapted from the British Society of Audiology (2011)]. The Wechsler Abbreviated Scale of Intelligence-Second Edition (WASI-II) (Wechsler, 2011; McCrimmon and Smith, 2013) was administered to assess intelligence, and all the participants were determined to have normal intelligence. More details of participants are shown in Table 1 and Supplementary Appendix I.

**TABLE 1:** Demographic information of participants.

Participants	Gender		Age (Range)	Implantation age (Range)	Duration of CI (Range)
	Male	Female			
NH	12	13	6; 0 (5; 1–7; 8)	\	\
CI	10	11	6; 8 (5; 0–10; 9)	3; 1 (1; 5–9; 7)	3; 7 (1; 2–5; 6)

Parents of all participants provided written informed consent prior to the experiment. The experiments were approved by the Ethical Committee of Sun Yat-sen Memorial Hospital, Sun Yat-sen University.

### Experimental Procedure

Before the start of the neural experiment, all participants were asked to respond to a set of questionnaires evaluating handedness, health state, and intelligence. CI participants additionally answered a questionnaire consisting of CI-related questions, such as the duration of deafness and the duration of cochlear device usage.

#### Neural Experiment

**FIGURE 1:**
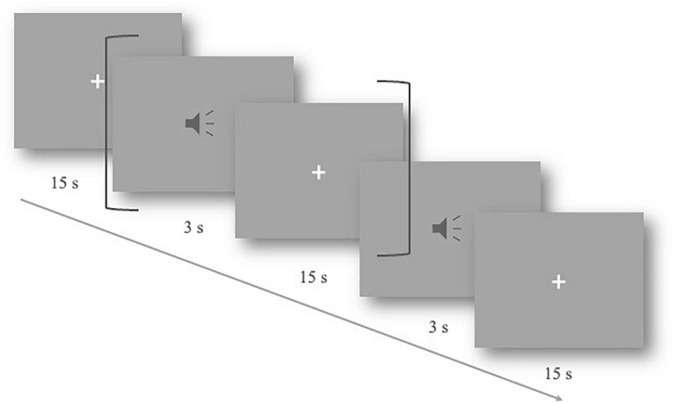
Slow event-related experimental design. One trial lasted 18 s, including 3 s of the auditory stimulus and a 15-s resting period (shown by dark gray brackets). The total procedure includes 40 trials.

The experiments were carried out in a quiet, shielded room. The participants were instructed to sit in front of the computer screen. After wearing the fNIRS cap, the signal quality of the channel formed between optodes was tested. During the experiment, the subjects were asked to keep still, relax, and listen to the auditory stimuli carefully. They were also informed that no response was required. The experimental materials were randomly played at 75 dB. After each stimulus, there was a 15-s resting period to allow for the hemodynamic response to return to baseline (Figure 1). The duration of the whole experiment was approximately 15 min.

**FIGURE 2:**
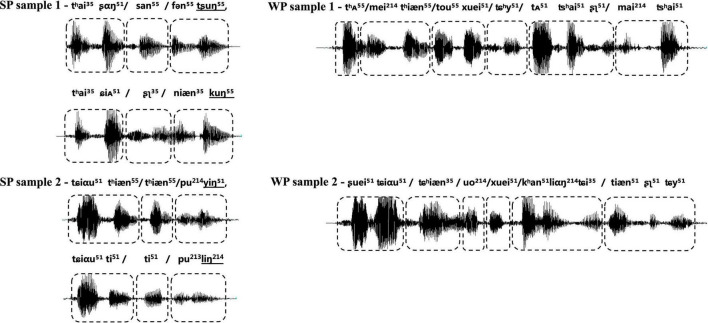
Waveform of samples from two types of experimental stimuli, with strong-prosodic (SP) in the left column and weak-prosodic (WP) in the right one. The boxes with dashed lines indicate the meters of the sentence. The shapes of sound waves show the parallel construction of rhythm and stress within each SP, together with parallel meters as indicated by the boxes with dashed lines; the ending syllables in the two parallel clauses rhyme with each other, as shown by the underlined international phonetic signs in SP samples.


Participants were exposed to an auditory task in an event-related format, which included two types of natural utterance stimuli (see Figure 2). The first condition contained natural SP sentences frequently used as proverbs in daily life. The proverb sentences have an identical rhyming scheme, and each one was formed by two clauses with parallel meters (indicated by the “/” in the following examples), and stress, as well as an identical number of syllables. The ending syllable in each clause rhymes with each other. With such acoustic and prosodic features integrated into one sentence, the SP sentences all have a strong sense of rhythmic harmony. Counterpart examples in English, though not as common as in Chinese, are “No pain, no gain,” “A friend in need is a friend indeed,” etc. Proverbs have fixed interpretations and are commonly used in daily life to express some sort of principles, knowledge, beliefs, etc. Here is an example of the usage of the proverb in Example (1): “You must practice more for this race, since ‘one minute on stage takes ten years of practice.”’

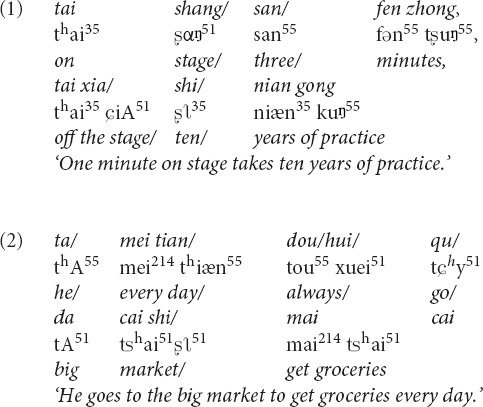


The second condition contained WP sentences, which are commonly used in daily conversations. The well-designed prosodic features existing in the SP were avoided in the WP condition, for instance, parallel meters and stress [refer to an example (2)]. Prosodic features in two types of stimuli were illustrated in the following figure of the waveform. Acoustic records of all the materials are available on https://pan.baidu.com/s/1vgiYoU_XqVd4vWRLSjA0VA?pwd=nirs.

No significant difference was found between the two groups of stimuli for the word frequency (mean: 1,820.43 vs. 2,094.60 pre-million, *p* = 0.20, based on Chinese Word Frequency Corpus, BLCU) to make sure every character occurs frequently in the daily life. Furthermore, 10 Mandarin Chinese-speaking university students and 10 children at the age of six were invited to grade the frequency of utterances heard and said in daily life by using a 5-point scale for this familiarity rating. The average scores of stimuli with and without SP features rated by university students were 4.90 and 4.80, respectively, while the average scores of children were 3.20 and 3.50, respectively. We also controlled the grammar of the sentences. The two groups of sentences shared five commonly used syntactic composition rules in parallel, including subject-predicate, verb-object, modifier-head, passiveness, and coordination. In addition, the semantics of sentences in both conditions were easy to retrieve due to their common usage.

Both conditions contained 20 trials of sentences. The experimental materials were recorded by a professional male announcer in standard Mandarin. Due to the slow event-related experimental design (for detailed denotations and explanations, refer to Aarabi et al., 2017) with longer intervals (15 s) between stimuli, there was no strict requirement for the duration of stimulation materials to be accurate to milliseconds. The audio was edited by Audacity to cut the blank segments, ensuring that the duration of the stimuli was 2–3 s, about 2.5 s on average. Only 100–500 ms of blanks in long pauses of the utterances caused by the announcer were cut so that the naturality of the speech sentences was well kept.

#### Functional Near-Infrared Spectroscopy Measurements

**FIGURE 3:**
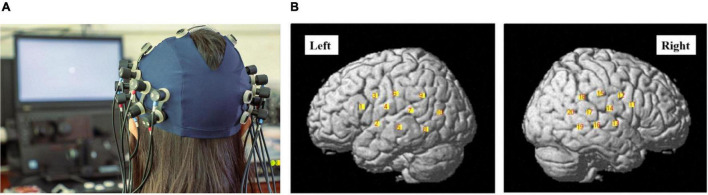
**(A)** The photograph of the optode array placed on the head of one of the participants (with consent). Red and blue labels on the optodes indicate optical sources and detectors, respectively. **(B)** The channel positions following registration to a brain atlas with different numbers (channels 1–10 left-hemisphere, channels 11–20 right-hemisphere).

Measurements were carried out with a total of 16 optodes arranged in two 2 × 4 arrays (each containing four sources and four detectors). The distance between the source and detector was set at 3 cm, and the optodes were positioned crosswise from each other. Hemodynamic responses were measured at the midpoints between the source and detectors, which were called “NIRS channels,” by the fNIRS imaging system (LIGHTNIRS; Shimadzu Co., Ltd., Tokyo, Japan). The arrays were placed on both sides of the head (Figure 3A), aiming primarily to measure cortical activation in the bilateral temporal cortex, inferior frontal cortex, and inferior parietal cortex.

Three different wavelengths (780, 805, and 830 nm), each with a pulse width of 5 ms, were used to calculate hemodynamic responses. The details of the head cap and the systems were described previously (Takeuchi et al., 2009; Takamoto et al., 2010; Takakura et al., 2011). After the recording, the three-dimensional (3D) locations of the optodes were measured by a 3D Digitizer (Nirtrack; Shimadzu Co., Ltd.) in reference to the bilateral tragus, nasion, and Cz. The measurement was performed by the “Spatial registration of NIRS channel locations” function of the NIRS-SPM Version 3 software, which is a SPM8- and MATLAB-based software package for the statistical analysis of NIRS signals (Ye et al., 2009). The channel positions are shown in Figure 3B.

### Speech Communication Ability Evaluation

The speech communication ability of participants was tested following Zhang et al. (2020)’s speech elicitation procedure. The procedure included three types of tasks: picture description, video content statement, and free conversation. In the 15-min test, children were asked to describe the content of two pictures, two videos, and freely talk about familiar personal experiences (e.g., family situations, favorite games, or cartoons).

Each participant’s test performance was scored by a specifically designed deep-learning framework (Zhang et al., 2020), which gave an evaluation of five major linguistic aspects of speech and language: pronunciation, expression efficiency, fluency, grammar, and semantics (Table 2). The overall speech communication ability score was calculated by averaging the five scores of their different linguistic aspects. The detailed scoring method is described in Supplementary Appendix II.

**TABLE 2:** Speech communication ability (SCA) evaluation scores on three levels.

Overall SCA	Scores on major linguistic aspects	Scores on the finest level of observation
**SCA total score**		
	PRONUNCIATION	Initial consonants accuracy
		Vowel accuracy
		Tone accuracy
	EXPRESSION EFFICIENCY	Syllable count Speech speed
		Pronunciation duration
	FLUENCY	Content restatement/replication
		Redundant articles
		Pause count
		Pause duration
	GRAMMAR	The wrong usage of grammar
	SEMANTICS	Key words missing

### Data Analysis

As Beta weight was reported to be a better parameter reflecting the activation level of certain cortex area to specific stimuli (Plichta et al., 2007; Ye et al., 2009) while oxygen-hemoglobin concentrations (HbO) provided information about how the activation level changed with time, we used both parameters to investigate activation patterns of SP stimuli. To explore how activation patterns differ for SP vs. WP stimuli in children with NH (the first research question), we compared the Beta weights, HbO concentrations, and functional connectivities between SP and WP conditions in the NH group. To verify our second hypothesis related to the abnormal pattern of prosodic perception in the CI group, comparisons between groups were conducted using Beta weights, HbO concentrations, functional connectivities, and laterality in response to SP and WP conditions. Furthermore, to investigate the relationship between neural responses to SP features and SCA (the third research question), we calculated the correlations between Beta weights of both conditions and SCA scores based on Pearson’s correlation.

#### Beta Weights Analysis Based on the General Linear Model

The present study focused on the changes in the oxygen–hemoglobin concentration, which has been reported to be sensitive to neuro-hemodynamic relationships (Hoshi et al., 2001; Strangman et al., 2002; Yamamoto and Kato, 2002).

Based on the general linear model (GLM), NIRS-SPM version 3 was used for analysis along with SPM 8 (Plichta et al., 2007; Ye et al., 2009). The time course of HbO was correlated with the design matrix using a boxcar function hemodynamic response function (hrf) to explain the data as a linear combination of an explanatory variable (Beta weight) plus an error term. After preprocessing with DCT detrending, hrf low-pass filtering, 128 Hz high-pass filtering, and Beta weights were obtained using an ordinary least square fit. More details were described by Ye et al. (2009).

Spatial activation maps of two groups under each condition were created using Beta weights and three contrast-model matrices in NIRS-SPM. The three contrast-model matrices were as follows: (1) SP condition [1 0 0] to examine HbO concentrations related to SP perception contrasting to the baseline, (2) WP condition [0 1 0] to examine HbO concentrations related to WP perception contrasting to the baseline, and (3) SP vs. WP condition [1 –1 0] to remove HbO concentrations common to both conditions and the non-task-related activation, which can be interpreted as the background of the brain activities.

**FIGURE 4:**
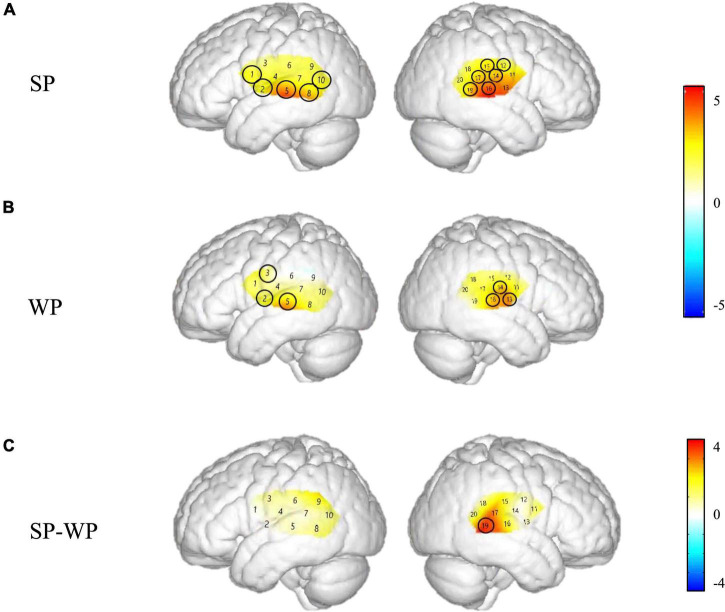
Cortical activation maps of the normal hearing (NH) group on the normalized brain surface. The color scales represent the *T* values, with statistically significant activated channels circled in black [*p*_*FRD–cor*_ < 0.05]. **(A,B)** Lateral views of significantly activated channels under strong-prosodic (SP) and weak-prosodic (WP) conditions contrasted against silence, respectively. **(C)** Lateral views of significantly activated channels by contrasting between conditions, indicating the stronger activated neural areas driven by the SP features in SP sentences.

**FIGURE 5:**
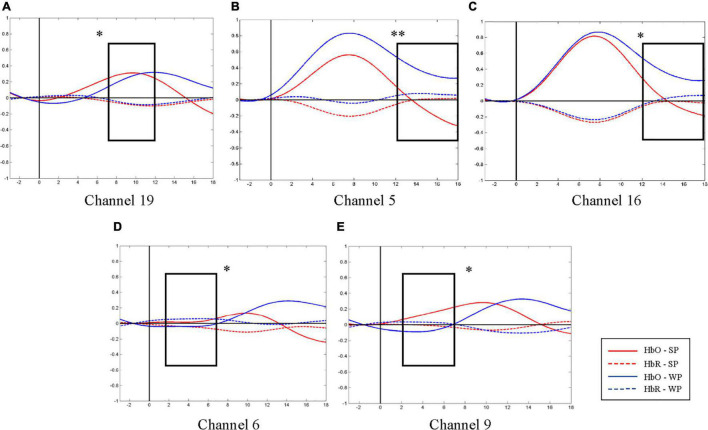
Average oxygen-hemoglobin concentrations (HbO) envelopes of the normal hearing (NH) group in the chosen time windows. **(A)** Channel 19 (the right middle temporal gyrus) had more significant activation in 7–12 s under strong-prosodic (SP) condition [*F*(1,48) = 4.086, *p*_*FRD–cor*_ = 0.049]. **(B,C)** Weak-prosodic (WP) stimuli evoked more significant activation in channels 5 [*F*(1,48) = 9.543, *p*_*FRD–cor*_ = 0.003] and 16 [*F*(1,48) = 4.398, *p*_*FRD–cor*_ = 0.041] in 12–18 s. **(D,E)** Channels 6 [*F*(1,48) = 4.358, *p*_*FRD–cor*_ = 0.042] and 9 [*F*(1,48) = 4.636, *p*_*FRD–cor*_ = 0.036] showed significant stronger responses to SP. *p < 0.05 and **p < 0.01.

**FIGURE 6:**
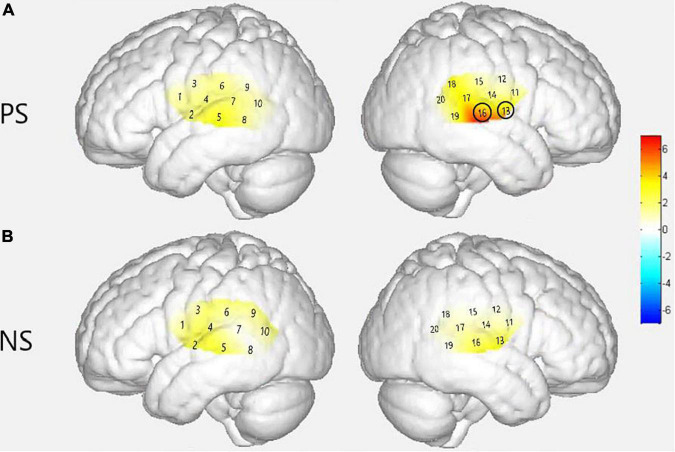
Group level cortical activation maps by contrasting between groups in the views of the normalized brain surface. The color scales represent the *F* values, with statistically significant activated channels circled in black [*p*_*FRD–cor*_ < 0.05]. **(A)** Lateral views of significantly activated channels in normal hearing (NH) group under the strong-prosodic (SP) condition than CI. **(B)** Lateral views of activating difference under the weak-prosodic (WP) condition by contrasting two groups of participants.

To explore how activation patterns differ for SP vs. WP stimuli in NH children, one sample *t*-test was used to create spatial maps of significant activation at the group level for each of these three conditions in the NH group as shown in Figure 4 (SP and WP conditions) and one-way ANOVA was calculated as shown in Figure 5 in two groups. We conduct the same analysis for the CI group and contrast activation map of both groups to give a general impress of the abnormal pattern of prosodic perception in the CI group. To further explore the activating difference between groups, one-way ANOVA was conducted under SP and WP conditions, respectively (Figure 6). Threshold images for the resulting group data were created using an alpha of 0.05. The value of *q* specifying the maximum false discovery rate (FDR) was set at 0.05. Areas of significant task-based activity are described in terms of cortical regions as opposed to cortical structures. The Beta values and the statistical results were visualized on spatial maps of the standard head model to indicate channels with significant results.

#### Event-Related Oxygen-Hemoglobin Concentrations

Event-related HbO concentrations provided information about how the activation level changed with time right after the stimulus began. Analysis of the data was performed in MATLAB (MATHWORKS, NATICK, and MA) using functions in the HOMER2 package (Huppert et al., 2009) together with custom scripts. The analysis included the following steps:

1. Exclusion of channels influenced by the external CI device. The hemodynamic responses of related channels could not be observed appropriately because the position of optodes overlapped with that of several participants’ external CI devices. Refer to Table 3 below for specific exclusions.

**TABLE 3:** The excluded channels.

Participants	Excluded channels
Subj_03	18
Subj_10	16, 17, 19
Subj_17	19, 20

However, other effective data of the channel gained from other subjects would still be retained. The data retention rate of all the channels was up to 98.6%, which did not affect the effectiveness of the overall data.

2. Conversion to optical density. The measured light intensity levels were converted to optical density using the HOMER2 *hmrIntensity2OD* function, a standard step in fNIRS data analysis (Huppert et al., 2009).
3. Motion-artifact correction. Motion artifacts were suppressed using the HOMER2 *hmrMotionArtifact* function.
4. Bandpass filtering. The optical density signals were bandpass filtered between 0.01 and 0.1 Hz by the *hmrBandpassFilt* function to attenuate low-frequency drift and cardiac oscillation.
5. Conversion to estimated changes in hemoglobin concentrations. Optical density was converted to estimated changes in the concentrations of HbO and concentration changes of deoxyhemoglobin (HbR) through the application of the modified Beer-Lambert Law (Huppert et al., 2009). A default value of 6.0 was used for the differential path-length factor at both wavelengths.

To gain the fine-grained observation, data were averaged further within four-time windows to make sure that the duration of every segment was the same as 5–6 s due to the slow change of hemodynamic responses, namely, –3–2, 2–7, 7–12, and 12–18 s. The starting point was set before the stimuli to examine the possible top-down control of attention and the anticipating effect. Among them, the first interval was set before and through the trial onset. This time window was regarded as a relatively stable phase right before any neural activity starts to respond according to hemodynamics (cf. Plichta et al., 2007).

To explore how activation patterns differ for SP vs. WP stimuli in NH children, HbO concentrations of NH group in different time windows were analyzed by one-way ANOVA with IBM SPSS Statistic Version 22.0 (IBM Corporation, Armonk, NY, United States). The Greenhouse–Geisser adjustment to the degrees of freedom was applied to all ANOVAs to correct for the violation of the assumption of sphericity. When significant interactions were found, *post hoc* tests were performed using tests for the simple effect of one-factorial ANOVA and/or Fisher’s protected least significant difference test. The level of statistical significance was set at *p* < 0.05. The value of *q* specifying the maximum FDR was set at 0.05 to make sure the false positive rate was no more than 5% on average in processing the HbO from multiple channels.

To explore our second hypothesis related to the abnormal pattern of prosodic perception in the CI group, we conduct the same analysis in the CI group. As we assumed that the CI group had some deficit in perceiving prosody features, we expected to find that significant differences between SP and WP conditions found in the NH group would not appear in the CI group. Then, we used one-way ANOVA to explore between group differences in different time windows under both conditions.

Furthermore, neural processing of speech and language has been widely found to have effects of lateralization (Belin et al., 1998; Gandour et al., 2004). To explore if the SP perception function is allocated to a specific hemisphere (related to the first research question), we calculated the laterality between the symmetrically matched brain regions in the left and right hemispheres (viewed as a channel pair) for the same condition between the two groups. The laterality index was calculated by the formula (Coito et al., 2015) below. Then, one-way ANOVA was employed to investigate between condition differences in different time windows in two groups.

$$L\,a\,t\,e\,r\,a\,l\,i\,t\,y=\frac{H\,b\,O\,\left(L\,e\,f\,t\right)-H\,b\,O\,\left(R\,i\,g\,h\,t\right)}{H\,b\,O\,\left(W\,h\,o\,l\,e\right)}$$

#### Analysis of Functional Connectivity

Functional connectivity (FC) was analyzed to evaluate participants’ cortical network activity while listening to utterances. The brain regions do not process information in an isolated way but work as a network to accomplish certain functions. Hence, FC can indicate the network patterns that possibly differ between conditions and groups.

All channels were included in the FC analysis. A bandpass Fourier filter (0.01–0.1 Hz) in the time series of HbO signals was used, and then the time series was further separated into segments (3 s before the stimuli and 18 s after the stimuli). Each condition (SP and WP) contained 20 segments. Each trial was separated into two time windows (0–9 s and 9–18 s) and the correlation was calculated for each window. We calculated mean coefficients for each pair of channels in each time window among all subjects. Group mean coefficients greater than 0.6 were mapped as significant FCs in each condition, considering that the correlation coefficient smaller than 0.4 increases false-positive rates and the coefficient greater than 0.7 results in FC density maps with lower sensitivity because of reduced dynamic range (Tomasi and Volkow, 2010; Wang et al., 2018).

For statistical comparison of the FCs among the regions with group mean coefficients greater than 0.6 in each condition, three-way repeated measures ANOVA (condition × region pair × group, hereafter RM-ANOVA) was performed in the 0–9 s time window and 9–18 s time window.

#### Statistical Analysis Between Hemodynamic Responses and Speech Behaviors

To explore the relationship between neural responses to SP/WP features and SCA development, we calculated the correlations between Beta weights of both conditions and speech communication ability scores based on the Pearson correlation coefficient. To determine whether there was a significant relationship in general, we examined the data of both groups as a continuum. This is due to the fact that all children, whether or not they have CIs, have different speech abilities.

## Results

### Neural Activities Based on Functional Near-Infrared Spectroscopy

#### Sensitive Neural Responses to Strong-Prosodic Stimuli

To explore if there is any sensitivity or selectivity of neural responses to SP speech, we compared the Beta weights, HbO concentrations, and functional connectivities between SP and WP conditions in the NH group.

The Beta weights were calculated to identify the brain regions that were engaged in processing SP and WP sentences. Channels 1, 2, 5, 8, 10, 11, 13, 14, 15, 16, 17, and 19 responded to the SP condition significantly while the WP condition yielded activation in channels 2, 3, 5, 13, 14, and 16 (refer to detailed statistical results in Supplementary Appendix III), which showed that SP activated a broader scope of the brain areas than WP in both hemispheres.

Figure 4 demonstrates the cortical activation for each stimulation condition at the group level. In the NH group, areas including the left superior temporal gyrus, middle temporal gyrus, fusiform gyrus, angular gyrus and right superior temporal gyrus, middle temporal gyrus, supramarginal gyrus, and subcentral area, responded to SP (Figure 4A), namely, channels 1, 8, 10, 11, 15, 17, and 19.

**FIGURE 7:**
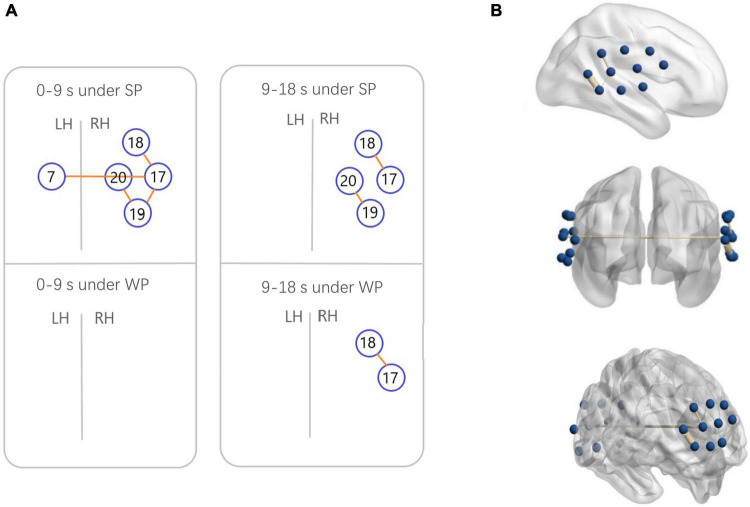
Functional connectivity (FC) maps among the activated cortical regions in the normal hearing (NH) group. **(A)** Blue circles indicate channels, and the orange lines show correlations greater than 0.6 (average across all participants). A significant correlation was found in Ch7-17 [*r* = 0.6231], Ch17-18 [*r* = 0.6222], Ch17-19 [*r* = 0.6194], and Ch19-20 [*r* = 0.6304] under strong-prosodic (SP) condition in time-window of 0–9 s. Under SP condition in time-windows of 9–18 s, significant correlation was found in Ch17-18 [*r* = 0.6561], Ch19-20 [*r* = 0.6088]. Correlation under weak-prosodic (WP) condition in time windows of 9–18 s was found in Ch17-18 [*r* = 0.6215]. **(B)** FC in the NH group under SP condition on the standard brain in time-windows of 0–9 s in different views. The blue dots indicate 20 channels, and the yellow lines show correlations greater than 0.6 (average across all participants).

Under WP, the NH group had more responsive areas in the left primary association cortex (channel 13), right primary auditory cortex, superior temporal gyrus, and middle temporal gyrus (channels 14 and 16), while the two groups shared the same activating areas of the bilateral middle temporal gyrus (Figures 4B, 7B).

As shown in Figure 4C, no significant main effect of conditions was found in any channel in the NH group (see details in Supplementary Table 3), but a marginally significant stronger response existed in channel 19 (right middle temporal gyrus) [*F*(1,48) = 3.917, *p*_*FRD–cor*_ = 0.054, η^2^ = 0.075], highlighting the sensitivity of the right middle temporal gyrus on processing SP sentences.

**FIGURE 8:**
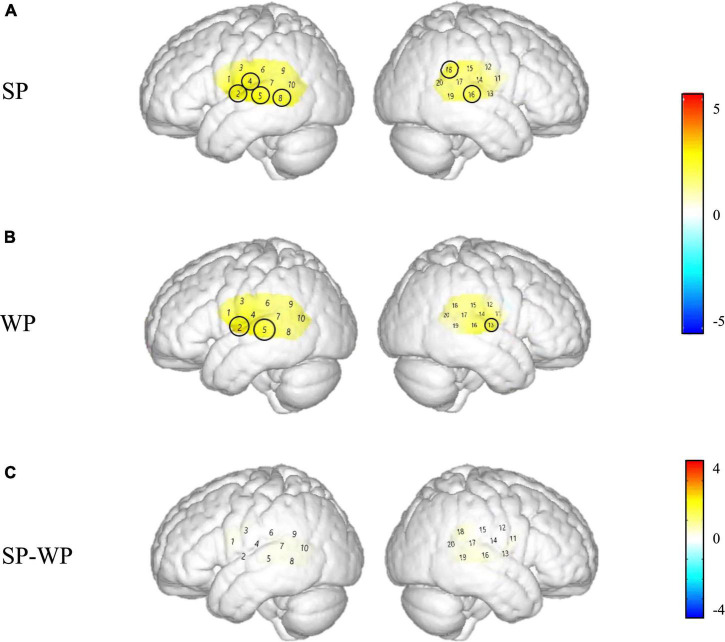
Cortical activation maps of the cochlear implantation (CI) group on the normalized brain surface. The color scales represent the *T* values, with statistically significant activated channels circled in black [*p*_*FRD–cor*_ < 0.05]. **(A,B)** Lateral views of significantly activated channels under strong-prosodic (SP) and weak-prosodic (WP) conditions contrasted against silence, respectively. **(C)** Lateral views of significantly activated channels by contrasting between conditions, indicating the stronger activated neural areas driven by the SP features in SP sentences.

**FIGURE 9:**
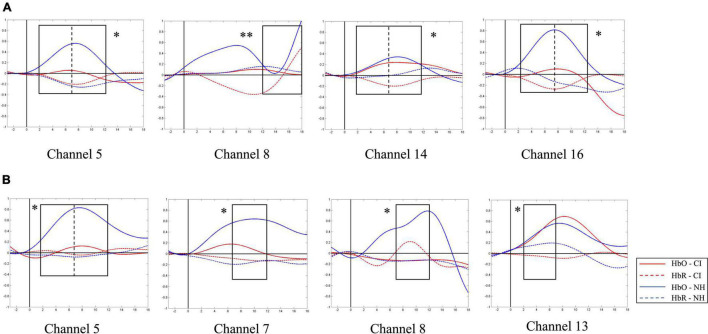
Between-group contrast of average oxygen-hemoglobin concentrations (HbO) envelopes under strong-prosodic (SP) and weak-prosodic (WP) conditions. **(A)** Hemodynamic responses to SP by both groups in channels 5, 8, 14, and 16. Higher activation by normal hearing (NH) group was found in time window 2–7 s in Ch5 [*F*(1,44) = 5.186, *p*_*FRD–cor*_ = 0.028], Ch14 [*F*(1,44) = 9.367, *p*_*FRD–cor*_ = 0.004], and Ch16 [*F*(1,44) = 10.162, *p*_*FRD–cor*_ = 0.003]; time window 7–12 s in Ch5 [*F*(1,44) = 5.084, *p*_*FRD–cor*_ = 0.029], Ch14 [*F*(1,44) = 9.736, *p*_*FRD–cor*_ = 0.003], and Ch16 [*F*(1,44) = 11.506, *p*_*FRD–cor*_ = 0.001]; and time window 12–18 s in Ch8 [*F*(1,44) = 8.994, *p*_*FRD–cor*_ = 0.004]. **(B)** Under WP, higher activation by NH group was found in time window 2–7 s in Ch5 [*F*(1,44) = 5.743, *p*_*FRD–cor*_ = 0.021], Ch13 [*F*(1,44) = 5.185, *p*_*FRD–cor*_ = 0.028]; and time window 7–12 s in Ch5 [*F*(1,44) = 4.483, *p*_*FRD–cor*_ = 0.04], Ch7 [*F*(1,44) = 5.932, *p*_*FRD–cor*_ = 0.019], and Ch8 [*F*(1,44) = 6.606, *p*_*FRD–cor*_ = 0.014].

**FIGURE 10:**
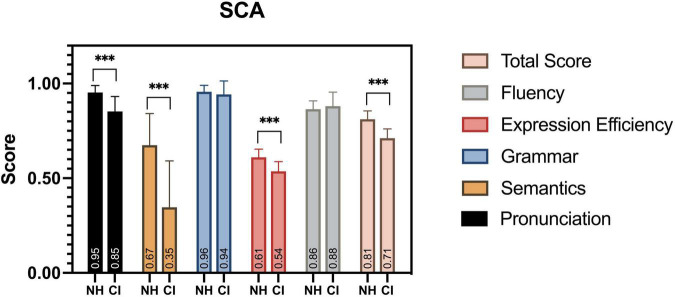
SCA assessment results. Between groups, significant differences in scores were found in total score, pronunciation, semantics, and expression efficiency. ***p < 0.001.

Furthermore, to characterize the HbO concentration in the time course under two conditions, we examined the difference within four time windows and the laterality in the NH group. A higher HbO concentration was observed under the SP condition than under the WP condition in earlier time windows: 2–7 s (channels 6 and 9, Figures 5D,E) and 7–12 s (channel 19, Figure 5A). Among these, the right middle temporal gyrus (channel 19) once again demonstrated its specialization in SP processing. In the later time window of 12–18 s, a higher activation under the WP condition was observed than under the SP condition in channels 5 and 16 (Figures 5B,C).

Laterality results showed that the cerebral activation was significantly right-lateralized when processing SP sentences in channel pair 9–18 in –3–2 s [*F*(1,48) = 1.324, *p*_*FRD–cor*_ = 0.04] and in channel pair 8–19 in 7–12 s [*F*(1,48) = 0.994, *p*_*FRD–cor*_ = 0.001], providing more evidence for the right-lateralization of SP processing in NH group.

Additionally, to test the cross-brain cooperation between different regions, we looked at the FC of two conditions in the NH group. Figure 7 illustrates pairs of regions with FC values greater than a threshold (*r* > 0.6) in each condition. For the NH group, there were high FCs throughout the trials among channels 17, 18, 19, and 20, which represented cortical regions around the right superior temporal gyrus, the middle temporal gyrus, the supramarginal gyrus, and the angular gyrus. Among them, the right middle temporal gyrus (channel 19) was involved in the broadest FCs. More connectivity results were found under the SP condition than under the WP condition (6 pairs vs. 1 pair), suggesting that SP processing required stronger FC between cortical regions which mainly occurred in the right hemisphere. Only one connection between the LH and RH was found under the SP condition but was not found in the WP condition during the first half of the trials.

#### Abnormal Neural Response Patterns of Cochlear Implantation Group

To verify our second hypothesis related to the abnormal pattern of prosodic perception in the CI group, we first conducted the same analysis in the Section “Sensitive Neural Responses to Strong-Prosodic Stimuli” for the CI group to see if what we found in the NH group would also be found in the CI group. We conducted direct comparisons between groups using Beta weights and HbO concentrations in response to SP and WP conditions.

As shown in Figure 8, between-condition contrast of Beta weights was also found in the CI group, with larger areas responding to SP than WP in channels 4, 8, 16, and 18 (refer to detailed statistical results in Supplementary Appendix III). In comparison, the CI group had a limited number of activated channels under the SP condition located only in a small area of the left superior temporal gyrus, the middle temporal gyrus, the subcentral area, the fusiform gyrus and the right superior temporal gyrus, the middle temporal gyrus, and the supramarginal gyrus (Figure 8A). As for between condition comparison in the CI group, no significant difference was observed (Figure 8C, refer to Supplementary Appendix III). Contrary to the different activation for the two conditions in the NH group, no significant main effect of condition was found within the CI group for HbO concentration. These results suggested that the CI group lacks neural sensitivity to process speech prosodic contrast.

Figure 6 demonstrated that responses between group comparison were significantly larger in channels 13 [*F*(1,44) = 5.259, *p*_*FRD–cor*_ = 0.027] and 16 [*F*(1,44) = 6.861, *p*_*FRD–cor*_ = 0.012] in the NH group compared with the CI group under the SP condition. There was no significant difference between the two groups under WP condition.

A similar between-group contrast was also found in HbO concentration. Under SP condition, the NH group generally had higher HbO concentration than the CI group throughout the time window of 2–12 s in channels 5, 14, and 16 (the left superior temporal gyrus, left middle temporal gyrus, right primary and association cortex, right superior temporal gyrus, and the right middle temporal gyrus) and the latest time window of 12–18 s in channel 8 (left middle temporal gyrus and fusiform gyrus) as shown in Figure 9A.

In addition, under WP condition, HbO concentration in the NH group was generally higher during the 2–12 s window (Figure 9B). However, there was no significant difference in –3–2 and 12–18 s time windows, but there were significantly different HbO concentrations in channels where the NH group always elicited stronger activation, including the left superior temporal gyrus, middle temporal gyrus, fusiform gyrus, supramarginal gyrus, and the right middle temporal gyrus (channels 5, 7, 8, and 13). It may be interpreted that children with CI had close-to-normal cortical processing for WP perception in the late phase of speech processing.

To analyze the statistical significance of the FC among the two conditions and the pairs of regions greater than the threshold, three-way RM-ANOVA (2 condition × 4 region pair × 2 group) was performed in the 0–9 s time window. A significant group effect was found [*F*(1,44) = 39.891, *p* < 0.001, η^2^ = 0.476]. However, no significant main effect {condition [*F*(1,44) = 2.552, *p* = 0.117, η^2^ = 0.055], region [*F*(3,42) = 0.770, *p* = 0.517, η^2^ = 0.052]} or interaction effect {group × condition [*F*(3,42) = 2.258, *p* = 0.140, η^2^ = 0.049], group × region [*F*(3,42) = 0.170, *p* = 0.916, η^2^ = 0.012], condition × region [*F*(3,42) = 0.558, *p* = 0.646, η^2^ = 0.038], and group × condition × region [*F*(3,42) = 1.906, *p* = 0.143, η^2^ = 0.120]} was found.

In addition, three-way RM-ANOVA (2 condition × 4 region pair × 2 group) was performed for the 9–18 s time window. A significant group effect was found [*F*(1,44) = 36.77, *p* < 0.001, η^2^ = 0.455]. No significant main effect {condition [*F*(1,44) = 1.716, *p* = 0.197, η^2^ = 0.038], region [*F*(3,42) = 1.697, *p* = 0.186, η^2^ = 0.107]} or interaction effect {group × condition [*F*(1,44) = 0.505, *p* = 0.481, η^2^ = 0.011], group × region [*F*(3,42) = 0.180, *p* = 0.909, η^2^ = 0.013], condition × region [*F*(3,42) = 0.960, *p* = 0.421, η^2^ = 0.046], and group × condition × region [*F*(3,42) = 0.033, *p* = 0.962, η^2^ = 0.007]} was found.

### Neuro-Behavioral Correlation

#### Speech Behavior Assessment

To address the hypothesis that neural responses to SP features are closely related to children’s speech communication ability, we probed all the participants’ SCA development by a behavioral test. Since abnormal neural activities were found in children with CI, we expected deficits to be found in their SCA assessment results.

All child participants in this study completed the SCA assessment, and significant differences were found at multiple observation levels of the evaluation (Figure 10). Children with CI were severely impaired in their speech development, with a significantly lower SCA total score, compared to their peers [*F*(1,38) = 0.895, *p* < 0.001]. The aspects of pronunciation [*F*(1,38) = 10.013, *p* < 0.001], semantics [*F*(1,38) = 5.706, *p* < 0.001], and expression efficiency [*F*(1,38) = 0.758, *p* < 0.001] had the most significant differences among the five levels of linguistic aspects. Similar deficits were also found in previous studies (Peng et al., 2004; Wu et al., 2008; Chen et al., 2017).

#### Neuro-Behavioral Correlation

We associated the scores of participants’ comprehensive speech communication ability with their hemodynamic performance represented by the Beta weights to further explore the connection between children’s neural activation of speech perception and their SCA. To determine whether there was a significant relationship in general, we examined the data of both groups as a continuum. The results of significant correlations under SP and WP conditions are shown in Tables 4, 5, respectively.

**TABLE 4:** Correlation between neural activation to strong-prosodic (SP) and evaluation scores of speech communication ability (SCA) test.

Channel	SCA total score	Pronunciation	Semantics	Grammar	Expression efficiency	Fluency
2						+ [*r* = 0.359, *p* = 0.023]
5	+ [*r* = 0.349, *p* = 0.029]		+ [*r* = 0.373, *p* = 0.018]		+ [*r* = 0.365, *p* = 0.021]	
7	+ [*r* = 0.385, *p* = 0.014]		+ [*r* = 0.392, *p* = 0.012]			
9						+ [*r* = 0.411, *p* = 0.008]
16	+ [*r* = 0.321, *p* = 0.033]	+ [*r* = 0.420, *p* = 0.007]				
17					+ [*r* = 0.336, *p* = 0.034]	
19	+ [*r* = 0.440, *p* = 0.016]		+ [*r* = 0.393, *p* = 0.012]		+ [*r* = 0.402, *p* = 0.01]	+ [*r* = 0.383, *p* = 0.015]

*+ symbol indicates positive correlation and − symbol refers to negative correlation.*

**TABLE 5:** Correlation between neural activation to weak-prosodic (WP) and evaluation scores of speech communication ability (SCA) test.

Channel	SCA total score	Pronunciation	Semantics	Grammar	Expression efficiency	Fluency
5	+ [*r* = 0.399, *p* = 0.011]		+ [*r* = 0.401, *p* = 0.01]			
16	+ [*r* = 0.338, *p* = 0.033]					
19					+ [*r* = 0.334, *p* = 0.035]	

*+ symbol indicates positive correlation and − symbol refers to negative correlation.*

More correlations were found under the SP condition than under the WP condition (14 vs. 4), suggesting that the perception of SP was more closely connected to SCA than the perception of WP. The overall level of SCA was predicted by cortical activation in channel 5 and channel 16 under both conditions. Activation in channel 19 (the right middle temporal gyrus) was sensitive to the largest range of evaluation scores (including the total score under the SP condition), which further verified its sensitivity to children’s speech development. The fluency score was related to the cortical activation data of the three channels under the SP condition, but no significant result was found under the WP condition.

## Discussion

We characterized the cortical responses to the perception of speech sentences with contrasting prosodic features in NH children and children with CI. In NH children, SP sentences evoked broader and right-lateralized cortical responses compared to WP stimuli. To our knowledge, this is the first time that the neural mechanism underlying the perception of natural catchy speech was investigated among children.

In addition, we identified deficits in cortical activation of SP speech perception in children with CI and examined the relationship between their speech behavioral performance and cortical responses. Consistent with previous findings (Luo and Fu, 2007), we observed significantly weaker activation in response to speech perception in the CI group. Moreover, more inhibited neural activities were found under SP than WP conditions for the CI group, suggesting that children with CI had a significantly impaired sensitivity to strong prosodic features of speech. Last, the neural responses to the SP sentences were found to correlate with the speech communication abilities of all the child participants.

### Strong-Prosodic Information Evoked Right-Lateralized Cortical Responses in the Early Processing Phase

Based on the direct comparison of Beta weights and FC results between SP and WP stimuli in the NH group, sentences with SP features evoked a larger brain network than speech with WP features (see Figure 8). Although previous studies on prosodic perception have found broad activation in both hemispheres (Gandour et al., 2004; Tong et al., 2005; Witteman et al., 2011), this is the first time that cortical responses to SP sentences are characterized in direct contrast to utterances with WP features.

As to the lateralization patterns, the results converge on the finding that SP sentences evoked right-lateralized activation and WP sentences activated left lateralized responses. For NH children, a higher HbO concentration was found during the middle time course in the right middle superior gyrus than that in the contralateral area (indicated by the laterality index in channel pair 8–19). An opposite result was observed between the identical pairs of the brain areas under the WP condition, which indicated the left lateralization of WP perception. The laterality index calculated on clustered data of each hemisphere also showed right-lateralized activation in the 7–12 s window in the SP condition. Furthermore, the connectivity results under the SP condition showed strong right laterality with five interhemispheric connectivities on the right and one bilateral connectivity. The different laterality tendency suggests the functional sensitivity of the right hemisphere for the auditory processing of slowly changing acoustic cues (i.e., slow F0 movements along the pronunciation of the whole sentences) that finally give a strong sense of utterance “catchyness.” This finding is in line with previous studies which found the specialization of RH on slow-changing prosodic signals (Meyer et al., 2002; Geiser et al., 2008).

Furthermore, the timewise HbO dynamics revealed selective responses to SP information in the early processing phases. In the first (2–7 s) and middle (7–12 s) time windows, higher activation to SP was found in the LH than in the RH (i.e., channel 19, Figure 7), which was consistent with the results of the Beta weights. In contrast, there was a higher HbO concentration in response to the WP condition than in the SP condition in both hemispheres in the late phase (12–18 s), suggesting a late selective response sensitivity to the speech with WP features. The FC results along the time course further proved this observation, with more significant pairs of connectivities observed under the SP condition in the early time window than in the late window (4 pairs vs. 2 pairs), and connectivity under the WP condition was only found in the late time window.

One possible reason for such time-wise imbalanced selectivity was that the rhythmic features in SP speech could be integrated in a short time and facilitate semantic retrieval. In EEG studies that had a high temporal resolution, prosodic information, such as rhyme congruity (Chen et al., 2016; Hahn et al., 2021) was shown to be processed as early as 200 ms after stimuli onset. Its temporal priority in the auditory speech processing was much higher than that of syntactic and semantic information which have been claimed to be processed in the late time window of 400–1,000 ms, i.e., widely recognized ERP components of N400 and P600 (Zhang et al., 2013; Delogu et al., 2019). Considering that the SP stimuli used in our study contained multiple features of prosody, with rhythm, meter, and stress being concordantly combined, such neural enhancement in the early phase of SP speech suggests that the integrated SP features in speech are processed quite fast.

The late higher cortical response in the WP condition was possibly caused by syntactic and semantic integration for sentence interpretation. Although we controlled the semantics of sentences being used in both conditions to include words and phrases commonly used in daily life, the SP sentences had relatively fixed syntactic structures (i.e., the paralleled phrases in each SP sentence use identical syntactic structure) in order to achieve a high level of prosodic harmony, which could facilitate the retrieval of the meaning of the sentence with an alleviative workload of integrating the syntactic and semantic information of the words inside (Martin, 1972; Bubic et al., 2010; Cason and Schön, 2012). Similar observations were also found in studies on formulaic speech perception (Wray, 2002; Jiang and Nekrasova, 2007; Millar, 2011). Extensive studies found that interpretation integration occurred later than phonetic perception (Friederici, 2002; Hickok and Poeppel, 2004; Zhang et al., 2019); hence, higher activation of WP was observed in the late window in trials.

### Children With Cochlear Implantation Are More Impaired in Their Neural Sensitivity to Strong-Prosodic Than Weak-Prosodic Sentences

There is well-established (human and animal) literature indicating that early exposure to sound/speech is vital for the proper development of the auditory system (Sharma et al., 2002; Kral and Eggermont, 2007; Kral, 2013), and children with severe-to-profound sensorineural deafness generally lack related experience. With a natural speech perception task in the present study, we found generally decreased activation in the CI group to both SP and WP than in the NH group, which is in line with previous findings. More importantly, characteristic deficits were found in processing SP stimuli, especially in that they showed a general absence of neural distinctiveness for SP vs. WP contrasts.

Inhibited cortical responses in the CI group were found in various ways when processing SP sentences. First, the CI group had particularly weaker activation in the right middle temporal lobe (channels 13 and 16), and no active connection was found in the CI group compared to the normal-hearing children showing as many as 7 pairs of activated FCs. The lower connectivities could result from the immaturity of these areas, as shown by the low activation level generally found in the CI group. Due to hearing deprivation, the function of the language-associated cortex was impaired and thus connection among these areas was still not formed, which might cause a deficiency in processing prosodic information.

Activation of auditory and auditory association brain regions by auditory stimuli after CI was found to be important to speech communication ability (Coez et al., 2008; Kral, 2013). Abnormal neural responses to speech (Lee et al., 2007; Feng et al., 2018b) and lower FC in the auditory tasks (Chen and Wong, 2017; Wang et al., 2021; Yusuf et al., 2021) were found in post-lingually deaf adults with CI. To our best knowledge, this is the first time to identify neural functional deficits in pre-lingually deaf children while perceiving natural SP sentences. Taking into consideration that preservation of neuroanatomical networks in auditory and auditory association brain regions in pre-lingually deaf children was related to better performance after CI (Lee et al., 2007; Feng et al., 2018b), the prohibited neural sensitivity to SP sentences identified here is possibly an important obstacle for the speech development of CI children. Functional recovery of these areas after CI is worth exploring with longitudinal studies for efficient speech rehabilitation.

Moreover, in the direct contrast of neural responses to SP vs. WP perception, different patterns regarding the laterality and time-wise HbO activation were found in NH children, but no between-condition difference was shown by the CI group, suggesting that children with CI had limited sensitivity to contrasting speech prosodic features. This finding was in line with previous studies that found that patients with CI had poorer F0 discrimination and showed a strong deficit in speech prosody perception (Marx et al., 2015; Jiam et al., 2017). Scholars also found that discrimination of lexical stress patterns in infants with CI was reduced compared with that of infants without CI (Segal et al., 2016); they also found that discrimination of lexical stress patterns in infants with CI was one of the prosodic cues that they could utilize in their first steps of speech acquisition. For the first time, our study provided neurological observations and the underlying neural deficits of such reduced prosodic feature sensitivity in pre-lingually deaf children with CI.

It is worth noting that although time-wise comparisons of HbO revealed that children with CI had generally decreased activation throughout the whole processing phase, no difference was found in the late time window of 12–18 s in WP condition, suggesting that in the late phase of speech processing, CI children had close-to-normal cortical responses to perceiving sentences with WP features. These results possibly indicate that syntactic/semantic integration abilities of children with CI were relatively well reserved when excluding the prosody processing requirement, and also further confirmed that the neural auditory development of pre-lingually deaf children was more impaired for perceiving speech prosodic features.

Additionally, we found no laterality effect on children with CI under two conditions, which was different from the right-lateralization under SP, left-lateralization under WP in the NH group. Considering all the children with CI were right-sided implanted, there is a possibility that the laterality of neural activities was influenced by the implantation laterality. However, there was a study suggesting that cortical processing of speech showed no influence on the implantation side in children (Wang et al., 2022). Thus, we postulated that the laterality of CI was not an essential factor for this result.

Considering the broad significant correlations to various aspects of SCA evaluation results (overall evaluation, pronunciation, semantics, expression efficiency, and fluency) found in the right superior and middle temporal gyrus, the neural sensitivity of this area to SP sentences plays an important role in SCA. It is promising to focus on this area in speech development for pre-lingual deaf people. The neural abnormalities regarding catchy utterance perception found in our study may be used to offer an objective assessment technique for young individuals with CI without speech foundations to evaluate speech-related neural development status and predict their rehabilitation outcomes. As speech perception abilities are the foundation for the development of speech expression (Curtin et al., 2017); it was hence worthy to explore if prosodic materials have a positive effect on speech training in the future.

### Children’s Neural Responses to the Strong-Prosodic Sentences Are Closely Related to Their Speech Communication Development

A striking finding in this study is that the neural responses to the SP sentences were widely correlated with the speech communication abilities of all the child participants. Compared to the WP condition, more activation regions in the SP perception were found to have a positive correlation with the SCA evaluation scores (Table 5). We speculate that children’s neural perception sensitivity to SP features in sentences is predictive of their speech development. Similar findings were found in a few behavioral studies (Falk, 2004; Wells et al., 2004; Nygaard et al., 2009; Herold et al., 2011). SP utterances (i.e., catchy sentences, sung speech, etc.) were found to enhance children’s working memory performance (Yuzawa, 2002; Roy and Chiat, 2004; Yuzawa and Saito, 2006), promote speech production (Adams and Gathercole, 1995; Baddeley, 2003), and facilitate speech acquisition (Mehler et al., 1988; Leong et al., 2017; Amichetti et al., 2021). The findings in our study highlight the significance of catchy utterance materials in the speech development of children. Nevertheless, the current results alone are unable to inform claims about the causal relationship between children’s SCA and their neural responses to prosodic perception. The wide range of correlations could plausibly stem from assortative processes involved in prosodic features in speech. The link may lie in that brain activities induced by catchy utterances help neural functional development, such as cortex maturation and FC formation. Future work could test this possibility by combining the forms of data collected in the current study with longitudinal studies to test the possible causality mechanism.

The right middle temporal gyrus (channel 19) was particularly sensitive to SP information as indicated by multiple results, such as significantly stronger activation measured both by Beta weights and HbO to SP than WP. HbO concentrations to SP were found to be significantly lateralized in this area compared to its contralateral part. Besides, broader connectivities were also found in this area, with significant connections to areas of the right middle superior gyrus, suggesting that the whole region around the right middle superior gyrus was particularly sensitive to SP perception. Most importantly, the response to SP in this area correlated to 4 items of SCA evaluation scores, including the overall score, semantics, expression efficiency, and fluency. As previous studies showed, the middle temporal gyrus was widely recognized as a part of the ventral stream in language processing, involved in mapping sound onto meaning (Hickok and Poeppel, 2004; Saur et al., 2008). The selective activation of this area for SP sentences suggests that natural catchy utterances might enhance the connections between prosodic auditory perception and semantic retrieval in children, which in turn accounts for the fast priming of neural processing for SP stimuli found in this study.

Past studies rarely focused on the integrated processing of natural catchy sentences with various prosodic features, as we focused on in the present work. The stimuli employed in previous studies were at the word level (Gandour et al., 1998, 2000; Hsieh et al., 2001; Li et al., 2003; Feng et al., 2018a), pseudo-sentences without semantic processing (Hurschler et al., 2013), or speech that only carries one aspect of prosody, such as intonation (Gandour et al., 2003; Tong et al., 2005), rhythm (Geiser et al., 2008), and stress (Tong et al., 2005; Sato et al., 2013). Our study provided the first insight into the neural responses responsible for the integrated processing of natural catchy sentences for children. This processing required a broader activation of the brain network in both hemispheres and was prominent in the area of the right middle temporal gyrus. Such processing patterns, as indicated by the close links to the speech behaviors, might be crucial for children’s speech development, and deficits in the neural processing would cause speech impairment, as discussed in the following section.

## Conclusion

We identified the characteristics of cortical responses to the perception of natural sentences with SP features in children. In NH children, SP sentences evoked broader and right-lateralized cortical responses than WP sentences. Stronger activation and functional connectivities were observed in the earlier phase of SP sentence processing, highlighting children’s neural sensitivity to integrated prosodic features of sentences. In addition, we identified more inhibited patterns in the perception of SP than WP utterance for pre-lingually deaf children with CI, manifested as less and weaker activation, lack of right-lateralization, as well as late response-onset and the abnormalities centered in the right superior and middle temporal gyrus.

Importantly, the neural activities to SP sentences were highly correlated with the speech communication performance of both normal and CI children, suggesting that neural sensitivity in speech prosody perception may be meaningful for children’s speech development.

The idiosyncratic neural responses to SP sentence perception in children with pre-lingual hearing loss shed light on the potential efficacy of SP utterances in their speech development, which is worthy of exploration in longitudinal studies.

Despite these insights, some limitations should also be recognized, aiming to provide opportunities for future work. First of all, the sample size in this study was small, with 25 individuals in the control group and 21 people with effective data in the CI group. Another limitation is the fact that we did not conduct actual longitudinal studies targeting the influence of SP sentences on children’s speech development and speech rehabilitation of children with CI. Meaningful results from such studies should be further interpreted in future studies and incorporated into clinical practice.

## Data Availability Statement

The datasets presented in this article are not readily available because of the privacy issues of clinical data. Requests to access the datasets should be directed to SL, lushuo@szu.edu.cn.

## Ethics Statement

The studies involving human participants were reviewed and approved by Ethical Committee of Sun Yat-sen Memorial Hospital. Written informed consent to participate in this study was provided by the participants or their legal guardian/next of kin.

## Author Contributions

YC and QL: experimental design, data collection, data analysis, and manuscript writing. ML and LG: data collection and data analysis. JY and JL: data analysis and manuscript editing. RF and YL: manuscript revising. GQ: experimental design and manuscript editing. YZ and SL: study co-supervising, experimental design, data analysis, and manuscript writing. All authors contributed to the article and approved the submitted version.

## Conflict of Interest

The authors declare that the research was conducted in the absence of any commercial or financial relationships that could be construed as a potential conflict of interest.

## Publisher’s Note

All claims expressed in this article are solely those of the authors and do not necessarily represent those of their affiliated organizations, or those of the publisher, the editors and the reviewers. Any product that may be evaluated in this article, or claim that may be made by its manufacturer, is not guaranteed or endorsed by the publisher.
