# Corrigendum: Children's neural sensitivity to prosodic features of natural speech and its significance to speech development in cochlear implanted children

**DOI:** 10.3389/fnins.2022.1007925

**Published:** 2022-08-24

**Authors:** Yuebo Chen, Qinqin Luo, Maojin Liang, Leyan Gao, Jingwen Yang, Ruiyan Feng, Jiahao Liu, Guoxin Qiu, Yi Li, Yiqing Zheng, Shuo Lu

**Affiliations:** ^1^Department of Otolaryngology, Sun Yat-sen Memorial Hospital, Sun Yat-sen University, Guangzhou, China; ^2^Department of Chinese Language and Literature, The Chinese University of Hong Kong, Hong Kong, Hong Kong SAR, China; ^3^School of Foreign Languages, Shenzhen University, Shenzhen, China; ^4^Neurolinguistics Teaching Laboratory, Department of Chinese Language and Literature, Sun Yat-sen University, Guangzhou, China; ^5^Department of Neurology, The Third Affiliated Hospital of Sun Yat-sen University, Guangzhou, China; ^6^Department of Clinical Neurolinguistics Research, Mental and Neurological Diseases Research Center, The Third Affiliated Hospital of Sun Yat-sen University, Guangzhou, China; ^7^Hearing and Speech Science Department, Guangzhou Xinhua University, Guangzhou, China

**Keywords:** natural speech perception, prosodic feature, neural response, cochlear implantation, speech communication ability, temporal cortex

In the published article, there was an error regarding the affiliation(s) for “Qinqin Luo.” As well as having affiliation 2, they should also have “School of Foreign Languages, Shenzhen University, Shenzhen, China.”

The authors apologize for this error and state that this does not change the scientific conclusions of the article in any way. The original article has been updated.

## Publisher's note

All claims expressed in this article are solely those of the authors and do not necessarily represent those of their affiliated organizations, or those of the publisher, the editors and the reviewers. Any product that may be evaluated in this article, or claim that may be made by its manufacturer, is not guaranteed or endorsed by the publisher.

