# Supplementary material for: Children’s Neural Sensitivity to Prosodic Features of Natural Speech and Its Significance to Speech Development in Cochlear Implanted Children
Source: Front Neurosci. 2022 Jul 12;16:892894. doi: 10.3389/fnins.2022.892894 (PMC9315047; doi:10.3389/fnins.2022.892894)
Supplement: Supplementary file 1 [file Data_Sheet_1.docx]

Supplementary Material

# Appendix Ⅰ

**Demographic information of participants**

| CI group | | | | | | | | | |
| --- | --- | --- | --- | --- | --- | --- | --- | --- | --- |
| ID | **Gender** | **Age** | **IA** | **Duration of CI** | **CI laterality** | **Hearing aids experience** | **Music learning experience** | **Literacy background** | **Dialect background** |
| Subj_01 | male | 8; 3 | 5; 7 | 2; 8 | unilateral; right | 2 years | none | RF | Cantonese |
| Subj_02 | female | 5; 4 | 1; 5 | 3; 11 | unilateral; right | none | none | RF | none |
| Subj_03 | female | 6; 1 | 2; 2 | 3; 11 | unilateral; right | none | none | RF | Cantonese |
| Subj_04 | female | 6; 1 | 1; 5 | 4; 8 | unilateral; right | none | none | RF | none |
| Subj_05 | female | 7; 11 | 3; 10 | 4; 1 | unilateral; right | none | none | RF | none |
| Subj_06 | female | 7; 10 | 6; 7 | 1; 3 | unilateral; right | 5 years | none | RF | none |
| Subj_07 | male | 6; 3 | 3; 1 | 3; 2 | unilateral; right | none | none | RF | Cantonese |
| Subj_08 | female | 5; 8 | 4; 4 | 1; 6 | unilateral; right | 1 year | none | RF | Cantonese |
| Subj_09 | male | 6; 10 | 3; 11 | 2; 11 | unilateral; right | none | none | RF | none |
| Subj_10 | male | 5; 0 | 2; 8 | 2; 4 | unilateral; right | none | none | RF | Cantonese |
| Subj_11 | male | 5; 2 | 1; 5 | 3; 9 | unilateral; right | none | none | RF | Cantonese |
| Subj_12 | female | 5; 4 | 2; 0 | 3; 4 | unilateral; right | none | none | RF | Cantonese |
| Subj_13 | male | 8; 8 | 3; 2 | 5; 6 | unilateral; right | none | 2 years | RF | Cantonese |
| Subj_14 | female | 7; 10 | 3; 0 | 4; 10 | unilateral; right | none | 1 year | RF | Cantonese |
| Subj_15 | male | 6; 0 | 3; 3 | 2; 9 | unilateral; right | 2 years | none | RF | Cantonese |
| Subj_16 | male | 5; 7 | 3; 1 | 2; 6 | unilateral; right | none | none | RF | none |
| Subj_17 | female | 8; 0 | 4; 10 | 3; 2 | unilateral; right | 2 years | none | RF | none |
| Subj_18 | male | 5; 5 | 2; 4 | 3; 1 | unilateral; right | none | none | RF | none |
| Subj_19 | male | 7; 11 | 4; 8 | 3; 3 | unilateral; right | none | none | RF | Cantonese |
| Subj_20 | female | 5; 1 | 3; 8 | 1; 5 | unilateral; right | none | none | RF | none |
| Subj_21 | female | 10; 9 | 9; 7 | 1; 2 | unilateral; right | 6 years | 3 years | RF | none |

*Note: IA stands for implantation age. RF stands for rehabilitation facilities.*

| ID | Gender | Age | Music learning experience | Literacy background | Dialect background |
| --- | --- | --- | --- | --- | --- |
| Subj_01 | male | 7; 1 | 2 years | 1^st^ Grade | none |
| Subj_02 | male | 5; 10 | none | KG | Cantonese |
| Subj_03 | male | 6; 8 | none | 1^st^ Grade | Cantonese |
| Subj_04 | female | 5; 4 | 1 year | KG | none |
| Subj_05 | female | 6; 1 | none | KG | Cantonese |
| Subj_06 | female | 5; 10 | none | KG | none |
| Subj_07 | male | 7; 2 | none | 2^nd^ Grade | none |
| Subj_08 | female | 5; 7 | 1 year | KG | none |
| Subj_09 | female | 6; 3 | none | KG | Cantonese |
| Subj_10 | female | 5; 9 | none | KG | Cantonese |
| Subj_11 | female | 6; 0 | none | KG | Cantonese |
| Subj_12 | male | 5; 4 | none | KG | none |
| Subj_13 | female | 5; 9 | 1 years | KG | none |
| Subj_14 | male | 6; 3 | 2 years | 1^st^ Grade | Cantonese |
| Subj_15 | male | 6; 0 | none | KG | Cantonese |
| Subj_16 | male | 5; 5 | none | KG | none |
| Subj_17 | male | 5; 3 | none | KG | none |
| Subj_18 | female | 5; 9 | none | KG | Cantonese |
| Subj_19 | female | 5; 2 | none | KG | Cantonese |
| Subj_20 | female | 7; 8 | 4 years | 2^nd^ Grade | none |
| Subj_21 | female | 6; 7 | none | 1^st^ Grade | Cantonese |
| Subj_22 | female | 5; 1 | none | KG | Cantonese |
| Subj_23 | male | 5; 5 | none | KG | Cantonese |
| Subj_24 | male | 6; 7 | none | 1^st^ Grade | none |
| Subj_25 | male | 7; 4 | none | 2^nd^ Grade | none |

*Note: KG stands for kindergarten.*

# Appendix Ⅱ

**Children Speech Communication Ability Evaluation**


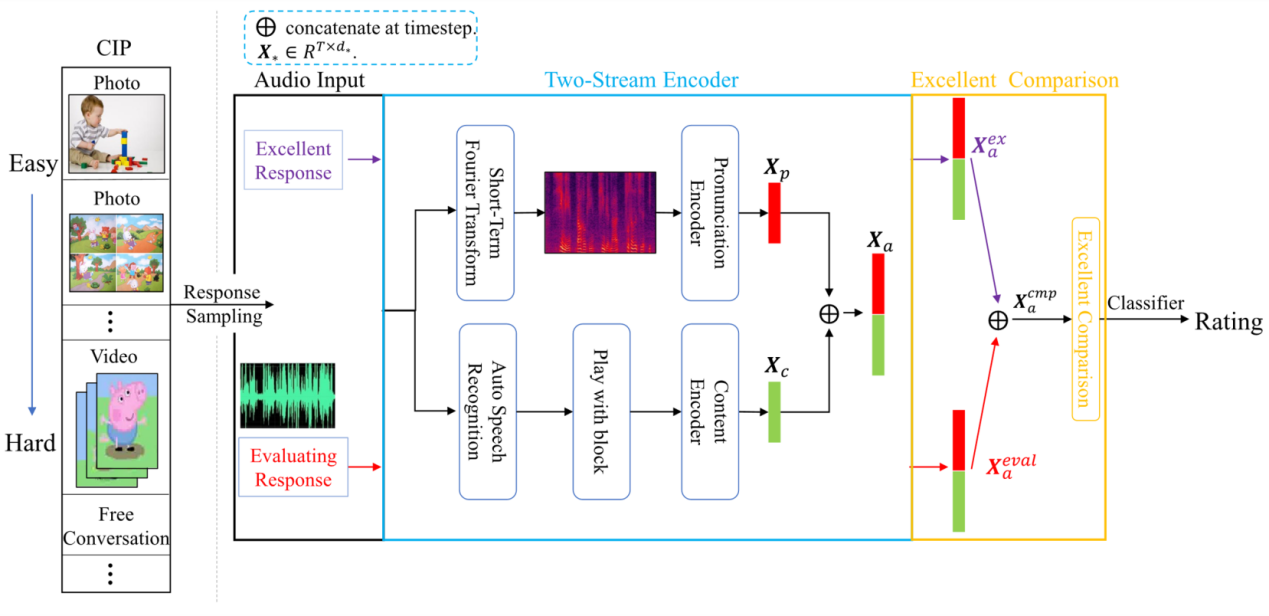


**Fig. S1 Children speech communication ability evaluation rating system (Zhang et al., 2020).** In the system, a comprehensive inducing procedure was design to collect children’s speech response firstly. Then, the evaluating response would be encoded by a two-stream encoder to extract the complementary feature from pronunciation and speech content for multi-aspect evaluation. Thirdly, the extracted features of the evaluating response and the excellent response (the sample with full score) were fed into a proposed excellent-comparison architecture. This excellent-comparison architecture was used to evaluate the response by comparison with the excellent response, by which the network was made more aware of the criterion of the procedure. Finally, the feature (after excellent comparison) was fed into a classifier to acquire the rating and the scores in the linguistic aspects of pronunciation, fluency, grammar were normalized as follows:

$$x^{'}=\frac{\max\{X\}-x}{\max\{X\}-min\{X\}} ,$$

where x∈X⊂R^N, X denotes all the scores (of different subjects) of a specific indicator of a rubric and N is the number of the subjects. For the indicators in the linguistic aspects of expression efficiency and semantic, the scores were normalized as follows:

$$x^{'}=\frac{x-min\{X\}}{\max\{X\}-min\{X\}} .$$

| Linguistic level | Linguistic aspect | Indicator | Definition and rating criteria |
| --- | --- | --- | --- |
| Low | Pronunciation | Initial consonant | Number of errors on initial consonants |
|  |  | Vowel | Number of errors on vowels |
|  |  | Tone | Number of errors on tones |
|  | Expression efficiency | Syllable count | Number of syllables |
|  |  | Speech speed | The number of syllables produced in a second |
|  |  | Pronunciation duration | The duration of the cumulative pronunciation |
| Medium | Fluency | Content restatement / replication | Restatements or repeated pronunciations |
|  |  | Redundant articles | Particles like “uh, a, then, this, that” being used as a gap filler between phrases or sentences |
|  |  | Pause count | Silence longer than 0.3 seconds counts as a pause |
|  |  | Pause duration | Cumulative duration of all pauses longer than 0.3 sec |
|  | Grammar | The wrong usage of grammar | Instances of incorrect grammar usage including of function words, grammatical construction, and word order |
| High | Semantic | Key words missing | Number of keywords missing and redundant words conflicting with the materials |

**Table S1 Rating criteria based on different Linguistic aspect**

# Appendix Ⅲ

**Statistic Results of Beta Weights**

| Condition | SP | | | | WP | | | |
| --- | --- | --- | --- | --- | --- | --- | --- | --- |
| Group | **Control** | | **CI** | | **Control** | | **CI** | |
|  | T value | Sig. | T value | Sig. | T value | Sig. | T value | Sig. |
| Channel 1 | **2.486** | **0.02** | 1.632 | 0.116 | 0.585 | 0.565 | 1.003 | 0.327 |
| Channel 2 | **2.174** | **0.04** | **2.276** | **0.032** | **2.577** | **0.018** | **3.155** | **0.005** |
| Channel 3 | 0.64 | 0.528 | **2.49** | **0.02** | 0.782 | 0.443 | 1.285 | 0.213 |
| Channel 4 | 1.929 | 0.066 | 0.873 | 0.391 | **2.311** | **0.031** | 2.003 | 0.058 |
| Channel 5 | **4.757** | **0** | **4.132** | **0** | **2.605** | **0.017** | **2.319** | **0.031** |
| Channel 6 | 1.433 | 0.165 | -0.26 | 0.797 | 1.634 | 0.117 | 1.355 | 0.19 |
| Channel 7 | 1.835 | 0.079 | 1.109 | 0.278 | 1.444 | 0.164 | 1.256 | 0.223 |
| Channel 8 | **3.312** | **0.003** | 1.634 | 0.115 | **2.207** | **0.039** | 1.036 | 0.312 |
| Channel 9 | 1.726 | 0.097 | -0.487 | 0.631 | 1.391 | 0.179 | 1.285 | 0.213 |
| Channel 10 | **2.061** | **0.05** | 1.417 | 0.169 | 1.207 | 0.241 | 1.557 | 0.134 |
| Channel 11 | **2.791** | **0.01** | 0.803 | 0.43 | 0.29 | 0.775 | -0.428 | 0.673 |
| Channel 12 | 1.106 | 0.28 | 0.339 | 0.737 | -0.23 | 0.821 | -0.398 | 0.695 |
| Channel 13 | **4.467** | **0** | **3.993** | **0.001** | 1.085 | 0.29 | **2.408** | **0.025** |
| Channel 14 | **3.297** | **0.003** | **3.399** | **0.002** | 1.303 | 0.207 | 1.513 | 0.145 |
| Channel 15 | **2.588** | **0.016** | 1.08 | 0.291 | 1.411 | 0.173 | 1.174 | 0.254 |
| Channel 16 | **5.279** | **0** | **3.307** | **0.003** | **2.126** | **0.046** | 1.7 | 0.104 |
| Channel 17 | **3.073** | **0.005** | 2.041 | 0.052 | 1.15 | 0.263 | 1 | 0.329 |
| Channel 18 | 1.203 | 0.241 | 1.766 | 0.09 | **2.339** | **0.029** | 1.218 | 0.237 |
| Channel 19 | **2.548** | **0.018** | -0.275 | 0.785 | 1.46 | 0.159 | 0.013 | 0.989 |
| Channel 20 | 1.286 | 0.211 | 0.494 | 0.626 | 1.266 | 0.219 | 0.757 | 0.457 |

**Table S2 Statistic result of Beta weights under two conditions**

|  | **NH** | | | **CI** | | |
| --- | --- | --- | --- | --- | --- | --- |
|  | ***F*** | **Sig.** | *η*^2^ | ***F*** | **Sig.** | *η*^2^ |
| **Channel 1** | 0.005 | 0.943 | 0.000 | 0.247 | 0.622 | 0.006 |
| **Channel 2** | 0.044 | 0.835 | 0.001 | 0.059 | 0.809 | 0.001 |
| **Channel 3** | 0.872 | 0.355 | 0.018 | 0.230 | 0.634 | 0.005 |
| **Channel 4** | 0.534 | 0.469 | 0.011 | 0.027 | 0.870 | 0.001 |
| **Channel 5** | 0.525 | 0.472 | 0.011 | 0.214 | 0.646 | 0.005 |
| **Channel 6** | 0.868 | 0.356 | 0.018 | 0.026 | 0.872 | 0.001 |
| **Channel 7** | 0.273 | 0.604 | 0.006 | 0.209 | 0.650 | 0.005 |
| **Channel 8** | 1.412 | 0.241 | 0.029 | 0.148 | 0.703 | 0.004 |
| **Channel 9** | 2.349 | 0.132 | 0.047 | 0.000 | 0.997 | 0.000 |
| **Channel 10** | 1.065 | 0.307 | 0.022 | 0.118 | 0.733 | 0.003 |
| **Channel 11** | 1.719 | 0.196 | 0.035 | 0.258 | 0.614 | 0.006 |
| **Channel 12** | 0.412 | 0.524 | 0.009 | 0.010 | 0.920 | 0.000 |
| **Channel 13** | 0.129 | 0.721 | 0.003 | 0.170 | 0.682 | 0.004 |
| **Channel 14** | 0.013 | 0.909 | 0.000 | 0.012 | 0.913 | 0.000 |
| **Channel 15** | 1.772 | 0.189 | 0.036 | 0.005 | 0.941 | 0.000 |
| **Channel 16** | 3.000 | 0.090 | 0.059 | 0.457 | 0.503 | 0.011 |
| **Channel 17** | 3.309 | 0.075 | 0.064 | 0.243 | 0.625 | 0.006 |
| **Channel 18** | 0.027 | 0.870 | 0.001 | 0.516 | 0.477 | 0.012 |
| **Channel 19** | 3.917 | 0.054 | 0.075 | 0.319 | 0.575 | 0.008 |
| **Channel 20** | 0.481 | 0.491 | 0.010 | 0.052 | 0.820 | 0.001 |

**Table S3 Statistic result of Beta weights contrasting between two conditions**
